# Navigating dementia care: a qualitative study of family care partners’ experiences in managing behavioural and psychological symptoms in Lima, Peru

**DOI:** 10.3389/frdem.2026.1774816

**Published:** 2026-03-16

**Authors:** Belen Custodio, Åsa Dorell

**Affiliations:** 1 Research Department, Instituto Peruano de Neurociencias, Lima, Peru; 2 Department of Neurobiology, Care Sciences and Society, Karolinska Institutet, Stockholm, Sweden

**Keywords:** behavioural symptoms, care partner support, dementia, dementia care, Latin America

## Abstract

**Introduction:**

Supporting a person living with dementia involves multiple challenges, particularly when responding to changes in behaviour and psychological symptoms, which can be emotionally demanding for care partners. Despite this, limited research has examined how care partners in Latin America experience and respond to these symptoms. Therefore, this study aims to gain an understanding of how family care partners of persons living with dementia experience and cope with behavioural and psychological dementia symptoms (BPSD).

**Methods:**

A qualitative study was carried out using semi-structured interviews with 17 family care partners of individuals with clinically confirmed dementia and BPSD, recruited from a private neurology clinic in Lima, Peru. Data were analysed using reflexive thematic analysis.

**Results:**

Three main themes were identified: (1) caregiving as an emotional and behavioural challenge, (2) the role of adaptation and flexibility when managing BPSD, and (3) building the care partner’s emotional well-being to support care. Care partners reflected on the challenges that arose from changes in behaviour in the people living with dementia, noting how these shifts sometimes influenced relationships and emotional well-being. In response, they employed adaptive strategies including modified communication, environmental adjustments, avoiding confrontation, and self-care.

**Discussion:**

Managing BPSD places a substantial emotional challenge on family care partners in Lima, Peru. Nonetheless, care partners actively develop strategies to respond to these situations. These findings contribute novel insights into dementia caregiving in underrepresented populations in Latin America and highlight the importance of understanding caregiving experiences in diverse cultural contexts.

## 1 Introduction

In recent years, dementia has been recognised as a major public health priority due to the exponential rise in cases worldwide (Alzheimer’s Disease International, 2009; Alzheimer’s Association, 2023; GBD 2019 Dementia Forecasting Collaborators, 2022). In 2019, an estimated 57.4 million people were living with dementia worldwide. This number is projected to increase by 166% by 2050, reaching 152.8 million cases (GBD 2019 Dementia Forecasting Collaborators, 2022). Latin America is one of the regions projected to experience a disproportionate rise in cases (GBD 2019 Dementia Forecasting Collaborators, 2022), and Peru is among the countries that clearly reflect this trajectory. In 2019, an estimated 196,699 people were living with dementia in the country, and this number is expected to rise to 744,847 by 2050, an increase of 279% (GBD 2019 Dementia Forecasting Collaborators, 2022). This increase not only places pressure on healthcare systems in terms of service utilisation and patient care, but also has significant social and economic implications, particularly in low- and middle-income countries (LMIC), where families of people living with dementia must navigate these challenges with limited formal support (Ibañez et al., 2020; Ibáñez et al., 2021).

Dementia is an acquired clinical syndrome characterised by a progressive decline in cognitive functions, affecting the person’s functionality and independence in performing daily living activities, with no current cure (Alzheimer’s Association, 2023; Ibañez et al., 2020; World Health Organization, 2021). Several causes of dementia have been identified, depending on the specific affected brain region (Alzheimer’s Association, 2023). The various forms of dementia commonly present, to varying degrees, with a combination of cognitive, functional, motor, and behavioural and psychological symptoms of dementia (BPSD) (Ibañez et al., 2020). The BPSD are increasingly recognised as core features of dementia and a cause of distress for the person who takes care and are closely linked to worse health outcomes for both the person living with dementia and their care partner (Bessey and Walaszek, 2019). It has been proven that 9 out of 10 people living with dementia will experience at least one BPSD during the course of the disease (Bessey and Walaszek, 2019). These symptoms are defined by the International Psychogeriatric Association as “symptoms of disturbed perception, thought content, mood, and behaviour frequently occurring in patients with dementia” (Draper et al., 2012), and can include delusions, hallucinations, agitation, aggression, depression, anxiety, apathy, disinhibition, irritability, motor disturbances, sleep disturbances, wandering, and inappropriate sexual behaviours (Kaufer et al., 2000; Kales et al., 2015).

A key actor in the care of the people living with dementia is the care partner, who assists them in performing basic and instrumental activities of daily living as cognitive decline progresses (Garcia-Ptacek et al., 2019). Two main types of care partners have been identified: formal and informal. A formal care partner is distinguished by receiving financial compensation through a contractual agreement and having professional training. In contrast, an informal care partner is typically a family member or someone within the patient’s close social circle who takes on this role without a formal contract or financial remuneration (García, 2010; Wimo et al., 2018). Unlike high-income countries (HICs), where individuals living with dementia are more likely to be transferred to nursing homes, in Latin America, most of the care provided to the people living with dementia is provided through informal care by unpaid family care partners, with women comprising the majority, who dedicate an average of 8–11 h per day to caring for their relatives (Ibáñez et al., 2021). A similar pattern is also observed in Peru, where a previous study found that 81.5% of care partners are women, and 60.9% of all care partners are the patient’s spouse (Custodio et al., 2014). This same study revealed a more complex and heterogeneous caregiving landscape, with caregivers ranging in age from 36 to 80 years, and their relationships to the person living with dementia varying between spouses (73.1%) and adult children (26.9%). The majority of these caregivers (84%) were married and faced competing demands, as approximately 38% continued working while caregiving, and nearly 69% reported reduced working hours due to caregiving responsibilities. Additionally, over 62% reported impacts on household economy, and many lacked adequate family and external support systems (Custodio et al., 2014). These varying family structures, life stages, relationship types, employment situations and socioeconomic pressures suggest that caregiving experiences may differ substantially depending on whether the care partner is a spouse, an adult child balancing filial duties with their own family and work obligations.

Care partners face a wide range of daily challenges associated with dementia, including cognitive decline, the need to assist their relatives with activities of daily living, and the management of BPSD (Isik et al., 2019). These ongoing demands often lead to what is known as caregiver burden, a multidimensional concept encompassing physical, psychological, social, and financial stressors related to the caregiving role (Ibáñez et al., 2021; Montesinos et al., 2024; Liu et al., 2020). Prior research has shown that caring for a person living with dementia imposes a greater burden compared to other illnesses (Alzheimer’s Association, 2023), with the BPSD being one of the main contributors to this burden (Jorge et al., 2021). It has been shown that care partners who deal with these symptoms daily are in a more vulnerable position (Isik et al., 2019; Montesinos et al., 2024; Ballard et al., 2000), as BPSD are unpredictable and often manifest in disruptive ways, making them particularly difficult to understand and manage (Cheng, 2017).

Living in an LMIC presents additional challenges due to barriers such as delays in accessing healthcare, unstable economies, and various social inequities (Ibañez et al., 2020; Ibáñez et al., 2021). Additionally, caregiver burden in Latin America and the Caribbean is among the highest globally, leading to poorer physical and mental health outcomes compared to care partners in other regions (Parra et al., 2018; Prince et al., 2012). A recent quantitative study conducted in Peru, using the Zarit Burden Inventory, has identified which BPSD placed the highest burden on care partners, identifying hallucinations, aberrant motor behaviour, and apathy as the most burdensome (Montesinos et al., 2024). However, little is known about how informal care partners in this setting experience these challenges in their everyday lives and what strategies they use to respond to these symptoms. Therefore, the aim of this study is to gain an understanding of how family care partners of persons living with dementia experience and cope with behavioural and psychological dementia symptoms.

## 2 Methods

### 2.1 Study design

A qualitative exploratory design was used, as we sought to capture an in-depth understanding of the unique and complex experiences of caring for a person living with dementia. The study was reported in adherence to the consolidated criteria for reporting qualitative research guidelines (COREQ) (Tong et al., 2007).

### 2.2 Study setting

The qualitative study was conducted in a private neurology clinic, called “Instituto Peruano de Neurociencias” (IPN), located in Lince, a central district of Lima, Peru, which is a reference neurological centre in the capital city. Peru is a multiethnic, middle-income country in South America and the third-largest nation by land area on the continent (The World Bank, 2025; Centro Nacional de Datos—CND, 2021). Its capital, Lima, has a population exceeding 10 million, accounting for 30.2% of the country’s total population (Instituto Nacional de Estadística e Informática—INEI, 2024). In 2024, at least one older adult (>60 years) resides in 45.8% of households in the capital (Ruiz-Calderón et al., 2024). Regarding dementia in Lima, a previous study reported a dementia prevalence of 6.9% (Custodio et al., 2008). The Peruvian healthcare system is highly fragmented, consisting of public and private sectors, each with multiple subsectors offering varying levels of coverage, funding sources, and service providers (Alcalde-Rabanal et al., 2011). This fragmentation results in inefficiencies in healthcare service delivery, limiting access for many Peruvians (Aguirre-Martens, 2023).

### 2.3 Participants and recruitment

Family care partners of people living with dementia experiencing BPSD were included. Eligible care partners were over 18 years old and provided care at least 3 days per week for a minimum of 8 h per day. Participants were recruited through non-probabilistic purposive sampling at the outpatient neurology department of the IPN. During clinic visits, the neurologist briefly introduced the study to eligible family care partners. Those who expressed interest were referred to the institution’s Research Department, where the study was explained in detail, including its purpose, voluntary nature, confidentiality, and participants’ right to withdraw at any time without affecting the care of their relative. Care partners who agreed to participate provided informed consent prior to the interview.

Guided by previous literature and the concept of “information power” (Malterud et al., 2016), an estimated 15–20 interviews were planned, considering factors such as sample specificity, study aim, theoretical framework, quality of dialogue, and analysis strategy. Between January 20, 2024, and February 28, 2024, the study was presented to 22 care partners, of whom 17 agreed to participate in the interviews. The five who declined did so due to lack of consent (*n* = 2), time constraints (*n* = 2), and a family emergency (*n* = 1). The three who declined due to time constraints or a family emergency were offered the opportunity to reschedule the interview at their convenience; however, none were able to accommodate an alternative interview date within the data collection period.

Table 1 presents the characteristics of the care partners and people living with dementia. Among the 17 care partners, 11 cared for someone with Alzheimer’s Disease (AD) and 6 for someone with Frontotemporal Dementia (FTD). Most had been primary care partners for 1–3 years (*n* = 14), while 3 had provided care for over 3 years. Additionally, 12 people living with dementia had been diagnosed within the past 3 years, and 5 for a longer duration. The people living with dementia cared for by these family care partners had varying levels of functional decline. Functional data were available for 15 people living with dementia. According to the Pfeffer Functional Activities Questionnaire (PFAQ) conducted by physicians, the majority exhibited moderate to severe functional impairment (PFAQ >7), while only one person living with dementia retained relatively preserved functional abilities (PFAQ <7). Regarding health insurance, 11 people living with dementia were affiliated with the social security system (EsSalud), 5 with the public health insurance (SIS), and 1 with private insurance. It is important to note that in Peru, all citizens without social security or private insurance coverage are affiliated with the public health insurance (SIS) (Alcalde-Rabanal et al., 2011). However, the health system in Peru faces significant challenges, including limited availability, extended wait times, and perceived quality concerns, which drive many individuals, regardless of their insurance type, to seek care at private institutions (Aguirre-Martens, 2023). Therefore, having any type of insurance does not exempt individuals from incurring out-of-pocket healthcare expenses. As recruitment was conducted at a private neurology clinic (IPN), this setting may have influenced the socioeconomic characteristics of the sample. The frequency of BPSD, as reported by physicians based on the Neuropsychiatric Inventory Questionnaire (NPI-Q) assessment (Kaufer et al., 2000), is presented in Table 2.

**Table 1:** Family care partners and people living with dementia characteristics.

Characteristics		Family care partners	People living with dementia
Age (years)	30–50	6	0
	51–70	7	5
	+70	4	12
Gender	Woman	14	11
	Male	3	6
Functionality (PFAQ)^a^	Mean (range)		17.9 (5–29)
	<7		1
	>7		13
Relationship with the person living with dementia	Spouse	7	
	Child	6	
	Other family member^b^	4	
Time caregiving (years)	1–3	14	
	+3	3	

a Pfeffer Functional Activities Questionnaire.

b Including siblings, nephews, among others.

**Table 2:** BPSD experienced by the person living with dementia, being cared for by family care partners.

Symptom	# of people living with dementia
Irritability	15
Delusions	14
Sleep disturbances	12
Agitation	9
Apathy	9
Hallucinations	4
Others	3

### 2.4 Data collection

Data was collected through in-person, semi-structured single interviews conducted in Spanish, to explore the participants´ experiences and how they cope with behavioural and psychological dementia symptoms.

These were audio-recorded and later transcribed verbatim by the author. The interviews were conducted at the facilities of the IPN, with only the interviewer and the care partner present in the consultation room to ensure the participant’s comfort. The interviews were conducted by the author BC, who is a Public Health researcher with prior training in qualitative methods and experience working with dementia care partners.

The initial questions focused on demographic data of the participants and the person living with dementia. The interview then focused on the challenges of managing BPSD and the strategies employed. Given the study’s primary aim to understand family care partners’ experiences and coping mechanisms in managing BPSD, other objective caregiving data, such as caregiver burden assessment scales or specific activities performed by the care partner, were not collected during the interviews.

Probing follow-up questions such as “Can you further describe…” and “Can you give an example of…” were used for clarification purposes. Interviews lasted between 38 and 74 min. The interview guide (Supplementary material), informed by prior research and clinical practice, was reviewed by an interdisciplinary team including a qualitative researcher, a psychologist, and a neurologist. Field notes were taken during interviews to support the dialogue; they did not contain additional information beyond the transcriptions. Before the interviews, physicians provided clinical data on the person living with dementia to help the first author tailor the interviews and characterise the care recipients of the participating family care partners.

### 2.5 Data analysis

The interview transcripts were analysed using reflexive thematic analysis (RTA), following framework Braun and Clarke’s (2006), with an inductive approach, allowing codes and themes to emerge progressively from the data. All the interviews were read by the first author (BC) to get an overall understanding of the data. Transcription, data organisation and initial coding were conducted in NVivo (v15.2), after which codes were exported to Microsoft Excel to support the development of overarching themes and sub-themes. Code similarities were explored through mind-mapping, which informed the creation of initial categories that were subsequently refined. Responses concerning the general caregiving experience were excluded from the analysis, as they were not directly related to BPSD and served primarily to establish rapport. Data were analysed as a complete dataset without stratification by dementia type or caregiver relationship with the person living with dementia. Coding and thematic development were led by the first author (BC) and further refined through iterative discussions within the research team to compare interpretations, examine similarities and differences, and enhance reflexivity and analytical rigour. To ensure trustworthiness, the authors (BC and AD) examined the coherence of the coding structure, consolidated data relevant to each code, and collectively agreed upon the final themes. Selected quotations were incorporated into the Results section to enhance transparency and credibility. Supplementary material provides an example of the analytical process.

### 2.6 Ethical considerations

All participants received detailed information about the study. Both verbal and written informed consent were obtained prior to data collection. Participation was voluntary, and participants were informed that they could withdraw from the study at any time. All collected data remained confidential and was anonymised. Regarding access to medical records, the authors did not access patients’ medical histories. Instead, treating physicians provided diagnostic and symptom-related information for each person living with dementia only after the care partner had provided informed consent and immediately prior to the interview. This information was shared with the explicit understanding that it would support the qualitative research and was managed in accordance with institutional data protection protocols.

Ethical approval was granted by the Ethical Board of the Hospital Nacional Docente Madre Niño San Bartolomé in Lima, Peru, with approval number 901-2024-0ADI-HONADOMANI-SB.

## 3 Results

Three main themes resulted from the analysis of the interviews. The first theme includes three sub-themes and focuses on the challenges that family care partners face when dealing with the BPSD. The second and third themes focus on how they encounter this process, with three and two sub-themes, respectively. The themes and sub-themes are presented in Table 3.

**Table 3:** Themes and sub-themes describing family care partners’ experiences in managing BPSD.

Themes	Sub-themes
Caregiving as an emotional and behavioural challenge	Changes in personality and behaviour
	The emotional weight of the BPSD
	Damaged caregiving relationship
The role of adaptation and flexibility when managing the BPSD	Changing the way of communicating
	Adapting the environment and routine
	Avoiding confrontation
Building the care partner’s emotional well-being to support caring	Taking time for myself is taking care of me
	Handling BPSD gives me calm

### 3.1 Caregiving as an emotional and behavioural challenge

This theme captures the challenges family care partners face in managing BPSD, particularly the behavioural and personality changes in their loved ones and the emotional impact of coping with these symptoms daily.

#### 3.1.1 Changes in personality and behaviour

Across interviews, care partners described the challenge of witnessing significant changes in the behaviour and personality of the person living with dementia. These changes were not experienced simply as symptoms, but as losses that altered the memory they had of their relative. Care partners repeatedly expressed a sense of bewilderment as their loved one became “a different person” for them, disrupting established relational roles and expectations at home.

*“I had observed a total change in my husband’s behaviour the previous year. He was always very loving. Very respectful. A gentleman, no? I mean, as a husband, he was perfect. And then all of a sudden, he changed completely in terms of behaviour.” (Participant 13)*

Even minor triggers could rapidly escalate, which care partners found emotionally draining and practically difficult to manage. Behavioural changes also created challenges beyond the home. Care partners felt responsible not only for de-escalating distressing behaviours but also for managing their social consequences, often apologising or explaining the situation to others.

*“We’ve had to ask several people to leave because she would confront them… No matter how much I told her, she made the decision. She decided to kick her out and did so… So, to avoid that confrontation, I had to say, ‘Madam, I’m sorry. My wife. She’s sick. Please…” (Participant 16)*

#### 3.1.2 The emotional weight of BPSD

Beyond the initial shock of behavioural changes, care partners described the sustained emotional toll of managing these symptoms daily. Feelings of sadness, helplessness, and frustration were common, especially when they felt unable to manage difficult situations or when the symptoms were directed at them.

*“She would get very anxious, she would cry and shout at me… and I felt very sad about it, and I also didn’t know how to manage at that time, eh? And well, that made me feel very, very frustrated.” (Participant 11)*

In several accounts, care partners also struggled emotionally when witnessing the distress of the person living with dementia, which intensified their own feelings of helplessness.

*“That made me shed a lot of tears, it made me feel very bad… I know that he was hurting himself too. It wasn’t just me, but also him. So that was the worst thing.” (Participant 13)*

Additionally, the emotional strain sometimes eroded care partners’ patience, leading to reactions they later regretted, followed by guilt and self-judgment.

*“I would like to go back in time and think that I shouldn’t have argued, but at the time, I said, but I am a human being, I got upset, just like everyone else…. You reach a point where you explode, don’t you?” (Participant 14)*

#### 3.1.3 Damaged caregiving relationship

Distinct from the more prevalent narratives of sadness and frustration, a less common but equally significant sub-theme was identified, the profound breakdown in their relationship with the person living with dementia, characterised by rejection, emotional distance, and relational rupture. These care partners expressed a deep desire to step away from the caregiving role due to emotional exhaustion.

*“I would really like to leave the house now… To leave her with, obviously not abandon her, but to leave her with the maid with a nursing technician during the day and one at night, and me to leave the home for a while, because I can’t stand it anymore. I really can’t.” (Participant 5)*

Despite this, they also felt guilt and moral responsibility, acknowledging that stepping back was not truly an option, as they remained responsible for coordinating and ensuring care. This sub-theme highlights how BPSD can challenge the core of the caregiving relationship itself, pushing care partners to the emotional limits of their role.

### 3.2 The role of adaptation and flexibility when managing the BPSD

This theme highlights the practical strategies family care partners developed and perceived as helpful to manage BPSD. The central focus of this theme is how family care partners had to approach their loved ones in a different way than they did before the onset of BPSD.

#### 3.2.1 Changing the way of communicating

As family care partners recognised that their relatives were exhibiting new behavioural patterns due to BPSD, many began to reflect on the need to modify how they communicated. The BPSD sometimes made their regular communication less effective, prompting family care partners to explore alternative strategies that incorporated non-verbal cues such as tone of voice, facial expressions, and physical gestures. For many, this was a gradual learning process shaped by everyday experiences.

*“To learn to communicate with her. No longer with words. But in other ways… I have learned over time. To use other methods of communication, for example in the way I touch her hand, the gesture, the face I make.” (Participant 8)*

This shift in communication style was often supported by a deeper understanding that their relative’s behaviour was a manifestation of the disease rather than a deliberate attempt to be difficult. This re-framing of intent appeared to be central to fostering more adaptive responses.

*“The truth is, you have to have a lot of patience; I had the shortest patience. But I have to be patient… because it is not that she wants to do it, it was because of her disease, and I understand that it is a disease and that she cannot overcome it, right?” (Participant 16)*

Through this evolving patience and the development of new ways of interacting, family care partners also highlighted the importance of regulating their own emotional responses, noting that their reaction directly influenced the person living with dementia’s behaviour.

*“We have to regulate ourselves so that she is also calm”. (Participant 10)*

#### 3.2.2 Adapting the environment and routine

Family care partners reflected on how attentiveness to small details in their relative’s behaviour and environment allowed them to manage episodes of BPSD in a better way. Rather than focusing solely on observable details, they described an active process of interpretation, what one participant metaphorically called “having a microscopic view”, to identify potential triggers.

*“He hugged him, and Person Living With Dementia 3 stayed (participant 3 imitated an uncomfortable face). His friend is very expressive; he speaks loudly. That’s when I realised that Person Living With Dementia 3 doesn’t like it when people talk loudly when people shout, or laugh loudly. No. That bothers him.” (Participant 3)*

The identification of such patterns allowed family care partners to make changes in the physical or social environment. These adjustments, such as removing a stimulus or modifying an interaction, were often intended to prevent or reduce the likelihood and intensity of the BPSD.

*“And what have I had to do? Hide the key. And now, the battery has been removed, and I even have the car outside the house… ‘He sees the car and he gets stubborn.” (Participant 6)*

Across these narratives, adaptation was not framed as a one-time solution or a fixed intervention, but as a dynamic and evolving process that required continuous re-evaluation.

*“We have found something that works, which I am aware may work now and probably won’t work later, and I will have to look for another one.” (Participant 10)*

#### 3.2.3 Avoiding confrontation

A common lesson care partners reported was that confronting or correcting the people living with dementia often made situations worse. Over time, they shifted toward avoiding conflict, disengaging from arguments, or redirecting conversations to maintain calm. This shift in approach was not framed as resignation, but rather as a form of adaptation of their reaction. Rather than challenging inaccuracies or delusional ideas, care partners prioritised emotional stability for both parties.

*“When I said again, ‘Go to sleep. She was angry. She said to me, ‘Thief, you’ve robbed me’. I didn’t answer her, because if I’d answered her, it’d be worse.” (Participant 2)*

In some cases, this adaptation to manage the BPSD included engaging with the person living with dementia’s perspective rather than contradicting it. Instead of confronting or correcting distorted perceptions, family care partners gently guided their relatives to realise things on their own, avoiding confrontation while still resolving the situation.

*“My mom says, ‘Let’s go buy fish.’ ‘But mum, it’s 19:00. Where am I going to get fish?’ I just follow her and I show the place where they sell fish. ‘You see? It’s already closed. Because it’s late’. I explain. ‘What time is it?’ she asked, and there I explain more or less ‘Mum, it’s late’… She sees the stand is closed and then she says ‘Ah it’s ok’, and we go back.” (Participant 4)*

Family care partners were aware of the need to avoid contradicting their loved one, as they felt it helped manage the BPSD. This strategy often involved a conscious weighing of outcomes. In moments of potential conflict, family care partners made choices that prioritised their own and people living with dementia’s calm over factual accuracy. Additionally, some family care partners described using distraction or redirection as a way to de-escalate difficult situations. These strategies included telling white lies or temporarily validating their beliefs. Though emotionally difficult at times, care partners explained that these approaches were reported as effective in avoiding distress and managing behavioural episodes.

*“My mom says, ‘I want to go home. My children are waiting for me, I have to go now’. And then I get sad sometimes when she says that to me, but what I tend to do at that moment is, ‘Yeah, well, mum, let’s go. Let’s go to your house’… so, I take her for a walk around the block or a walk in the park. I make her forget, and then I take her home. I say, ‘Here’s your home. This is your house.’ And she comes in, she forgot. And then, well, she comes into the house.” (Participant 4)*

### 3.3 Building the care partners’ emotional well-being to support caring

This theme highlights emotional well-being as both a personal need and a crucial resource that enables and sustains caregiving. Family care partners viewed emotional balance as an essential tool for managing BPSD and enabling other strategies, serving as the foundation that makes caregiving sustainable.

#### 3.3.1 Taking time for myself is taking care of me

Family care partners repeatedly expressed that taking care of their own emotional well-being directly impacted their ability to care well. Their well-being was seen as something that shaped their interactions and the success of the strategies described earlier. When overwhelmed or exhausted, their ability to stay calm and manage BPSD effectively declined:

*“Because when you get overwhelmed or tired, the care is not the same.” (Participant 2)*

Self-care was not limited to taking breaks from caregiving tasks; it also involved finding emotional release through conversation and connection. Some care partners highlighted the value of talking openly about their feelings with someone they trusted, while others described professional psychological support as a crucial space to be heard. These underscore how important it was for care partners to have a safe space to express their emotions, process their struggles, and feel acknowledged.

*“The psychologist there is the one who is supporting me. As I said to the doctor, ‘I thank you because during all this time you are the only person who is thinking about the caregiver’. I understand that they focused on my husband, but they didn’t ask me how I was feeling or what was going on with me. And with the doctor, I am in therapy. It helps me.” (Participant 6)*

#### 3.3.2 Handling BPSD gives me calm

While caregiving can be an emotionally stressful experience, care partners also described how successfully managing these symptoms brought them reassurance and a sense of control. Effective de-escalation not only improved the people living with dementia’s behaviour but reinforced care partners’ confidence in their own abilities:

*“It gives me a bit of calm and a bit of satisfaction. I say ‘I did well. I was able to control her’, Eh? It makes me feel good.” (Participant 4)*

In the end, managing BPSD was not perceived as just a technical skill; it became a feedback loop reinforcing the care partner’s sense of efficacy. When care partners see their strategies working, it brings emotional relief and motivates them to keep going. This sub-theme also reveals how important it is for care partners to feel they are managing situations well, showing how emotionally invested they are in doing things right.

## 4 Discussion

This study examined how family care partners of people living with dementia in Lima, Peru, experience and manage BPSD, focusing on their everyday strategies. Findings highlight the significant emotional and behavioural challenges involved, the need for flexible and adaptive responses, and the central role of self-care in enabling effective symptom management.

In this study, managing BPSD was emotionally challenging for family care partners, leading to exhaustion and, in some cases, the deterioration of the caregiving relationship itself. This finding aligns with existing quantitative and qualitative studies that highlight the emotional and behavioural toll of caregiving in dementia (Engel et al., 2022; Ayalon et al., 2025; Armstrong et al., 2022; Rathnayake et al., 2019; Ang and Yuen, 2023; Almberg et al., 1997). A major challenge reported by care partners was managing personality and behavioural changes in the people living with dementia. This is in line with findings from Australia, where such changes significantly impacted care partner well-being (Engel et al., 2022), and from studies involving both family and migrant care partners, who identified irritability and changed behaviour as particularly stressful (Ayalon et al., 2025). Similarly, care partners in Singapore described behavioural symptoms as their most difficult caregiving challenge (Ang and Yuen, 2023). Care partners also struggled to accept the change in their relatives’ behaviour, reflecting similar findings from another study where behavioural and cognitive changes were particularly distressing for the family (Burley et al., 2023).

The emotional impact of managing BPSD emerged as another relevant challenge, consistent with findings from diverse cultural contexts (Engel et al., 2022; Ang and Yuen, 2023; Owokuhaisa et al., 2023; Feast et al., 2016; Nguyen et al., 2024; Tu et al., 2022). Care partners in Asia reported feelings of helplessness, frustration, sadness, and emotional exhaustion when facing symptoms they struggled to manage (Nguyen et al., 2024; Tu et al., 2022). Similar emotions were expressed by care partners in this study, who described feeling overwhelmed by daily demands. A systematic review and meta-ethnographic synthesis further support these findings, noting that BPSD can erode communication and relational closeness, leaving care partners feeling unloved and distressed (Feast et al., 2016).

Beyond emotional strain, care partners in the present study also reported a deterioration in their relationship with the person living with dementia, often driven by experiences of rejection linked to BPSD. This perceived loss of reciprocity negatively affected the caregiving bond, as care partners felt obliged to provide care despite being treated with contempt. These results are supported by prior research showing that BPSD can erode care partner–person living with dementia relationships (Feast et al., 2016; de Vugt et al., 2003; Boughtwood et al., 2011). For instance, de Vugt et al. (2003) found that spousal care partners experienced relationship declines due to communication barriers that hindered emotional expression, reinforcing a sense of relational imbalance.

Despite the challenges described above, interviewees highlighted that being flexible and adapting to the situation can help manage BPSD more effectively. A key strategy involved adapting communication styles, being more patient and understanding that their loved one’s symptoms were a result of the disease. These findings align with other studies, where care partners explained that attributing BPSD to the illness, rather than to intentional behaviour, allowed them to approach the situation with greater empathy (Ayalon et al., 2025; Burley et al., 2023; Meyer et al., 2023). In addition to communication, participants also described adjusting the environment and daily routines of the person living with dementia. This is consistent with findings by Engel et al. (2022), where establishing a routine was seen as a coping mechanism. However, care partners in that study also emphasised that while having a routine is important, it is equally crucial to remain flexible, given the unpredictable nature of dementia symptoms. Another strategy involved avoiding confrontation as family care partners from Lima, Peru, observed that attempting to change their relative’s mind or arguing often escalated the situation. Studies in Israel have reported similar findings, where care partners described avoiding unnecessary conflict, especially over trivial matters, recognising that it was not beneficial for either the person living with dementia or themselves (Ayalon et al., 2025). A separate study conducted in the United States, which included African American, Latino, Asian, and American family care partners, found that they used techniques such as therapeutic fibbing or redirecting the person living with dementia’s attention to other activities to avoid conflict (Meyer et al., 2023). These same strategies were also reported by family care partners in the present study.

A noteworthy insight regarding the strategies developed was the emphasis on strengthening care partners’ emotional well-being. Interviewees consistently acknowledged that they needed to be well themselves to adequately care for their loved ones. This narrative has been previously reported in multiple studies (Engel et al., 2022; Ang and Yuen, 2023; Meyer et al., 2023). Engel et al. (2022) found that care partners recognise the importance of prioritising their mental health, knowing it would ultimately benefit the person living with dementia. Some suggested that practising self-care could make the difference between being an effective care partner and becoming overwhelmed (Malterud et al., 2016). Similarly, in a study conducted in Singapore, self-care was identified as a facilitator in making caregiving more sustainable (Ang and Yuen, 2023), a view also echoed by participants in the present study. Notably, across these studies and in the current one, care partners did not frame self-care as an individual benefit, but rather as something that directly benefited their loved ones. However, it is important to note that the well-being and emotional support strategies identified in this study were largely initiated and resourced by the care partners themselves, rather than provided as a benefit from the neurology clinic. While some participants accessed psychological support and others engaged in recreational activities, such resources are not universally accessible, particularly for those with limited financial means. This highlights a critical gap; the development of these coping strategies appeared to depend largely on care partners’ individual initiative and access to support systems, underscoring the need for healthcare systems to integrate accessible, structured psychosocial support and caregiver education programs that extend beyond the individual caregiver’s resourcefulness and are available regardless of socioeconomic status.

The findings of this study can be interpreted through the lens of the Transactional Model of Stress and Coping, which posits that stressful experiences are shaped by individual and environmental characteristics, and that people respond to stressors differently depending on these factors (Lazarus and Folkman, 1984). This model distinguishes between problem-focused coping, aimed at addressing or modifying the stressor, and emotion-focused coping, aimed at regulating the emotional response to it. In the context of dementia caregiving, family care partners face continuous stressors related to managing BPSD. Our results show that care partners employed multiple coping strategies, including modifying the environment, adapting communication, avoiding confrontation, and taking time for themselves. Most strategies were problem-focused, while efforts to preserve their own emotional well-being aligned with emotion-focused coping, highlighting the dynamic transactional process described by the model. Caregiving contexts are shaped by personal beliefs, values, and context, making it unlikely that a single coping approach would prove universally effective. Rather, the perceived appropriateness and effectiveness of a strategy depend on the specific context and characteristics of the family care partner and the person living with dementia (Owokuhaisa et al., 2023; Lazarus and Folkman, 1984; Baharudin et al., 2019). This complexity may help explain mixed findings in the literature. For instance, while a study in the United Kingdom found that emotion-focused coping was associated with better mental health outcomes among family care partners (Lloyd et al., 2019), another study in Malaysia reported that care partners using problem-focused strategies had a better experience caring for a person living with dementia (Baharudin et al., 2019).

### 4.1 Strengths and limitations

This study presents several strengths that contribute to the quality and originality of its findings. First, the source of diagnostic and symptom-related information was reported by neurologists, based on internationally recognised diagnostic criteria for dementia (Sachdev et al., 2014). This clinical validation strengthens the study by ensuring that the experiences described pertain to family care partners of individuals with clinically confirmed dementia and BPSD. Second, the study included a diverse sample in terms of caregiving relationships and dementia types, which allowed for a variety of perspectives. Although all participants were recruited from a single clinic in Lima, the sample included spouses, adult children, and other relatives, as well as family care partners of people living with dementia diagnosed with AD and FTD. In addition, this study addresses a significant gap in the literature, as to our knowledge, it is the first qualitative study conducted in Latin America exploring the lived experiences and strategies of family care partners managing BPSD, contributing context-specific insights essential for developing culturally appropriate and effective support strategies for care partners in the region. Nonetheless, several limitations should be acknowledged. First, participants were recruited from one private clinic in Lima. While efforts were made to ensure variability in care partners’ characteristics, and although the IPN is the first referral centre for dementia in Peru, participating in regional initiatives (Custodio et al., 2022; Ibanez et al., 2021; Crivelli et al., 2023), the study did not include care partners who rely solely on the public health system and cannot afford an out-of-pocket expense in a private clinic, or who live in rural areas. Differences in access to healthcare and socioeconomic conditions may shape distinct caregiving experiences and coping mechanisms. Another limitation is the gender distribution of participants, as most family care partners were women. While this reflects global and regional trends, the study may not fully capture the experiences of male care partners. Additionally, although the study collected data on care partners’ relationships with the person living with dementia, dementia type and time since diagnosis, the analysis did not stratify findings by these factors. This limits the ability to explore how the caregiving experience may differ between adult children caring for parents versus spouses or other relatives, or how BPSD manifestations and caregiving strategies may vary across dementia subtypes or disease progression stages. Lastly, another potential limitation is that coding was conducted by a single researcher, which could introduce coder bias. To mitigate this, several trustworthiness strategies were implemented (Shenton, 2004; Guba, 1981): credibility was strengthened through respondent validation during interviews and inclusion of illustrative quotes; transferability was enhanced through a diverse sample and rich contextual details; dependability was addressed through detailed methodology documentation and continuous peer debriefing; and confirmability was maintained through reflexive journaling and transparent coding decisions.

### 4.2 Implications for practice, policy and research

The findings from this study provide valuable insights that can inform the development of dementia services that are sensitive to the sociocultural realities of Peru and similar Latin American contexts. For example, healthcare services could consider integrating care models that acknowledge the central role of family care partners. Multidisciplinary teams within neurology clinics might provide not only medical management but also education and psychological support. Given that participants relied heavily on informal support networks and trial-and-error learning, training programmes could be prioritised to offer practical guidance on managing BPSD, including de-escalation techniques, environmental modifications, and recognising when professional help is needed. Importantly, such interventions must be evidence-based, culturally adapted, and grounded in theoretical frameworks, such as the Transactional Model of Stress and Coping in dementia research, which can guide the development of tailored support strategies that address both care partners’ interpretations of stressors and their coping resources, such as problem-solving skills, emotional regulation, and social support (Lazarus and Folkman, 1984).

From a policy perspective, this study underscores the need for governmental recognition of family care partners as key stakeholders in the dementia care continuum. Currently, Peru lacks an implemented national dementia plan that addresses both persons living with dementia and their family care partners (Sosa et al., 2024). Policymakers should consider integrating care partner support provisions into the national health agenda, alongside economic support mechanisms and capacity building in primary care settings.

Lastly, this study opens several opportunities for further investigation. Comparative research is needed to understand how caregiving experiences vary across different dementia types, disease progression stages, relationship with the person living with dementia, socioeconomic strata, geographic regions, and healthcare settings in Peru and Latin America. Studies in public hospitals, rural communities, and among lower-income populations would provide a more complete picture of dementia caregiving in resource-limited contexts. Additionally, research should explore the experiences of underrepresented care partner groups, including male care partners, adult children balancing multiple roles, and care partners of persons with less common dementia subtypes. Mixed-methods approaches that combine qualitative interviews with quantitative measures, such as caregiver self-efficacy scales, quality of life questionnaires, health literacy assessments, and social support instruments, could provide additional perspectives on caregiving outcomes and burden. Such evidence would strengthen the foundation for building sustainable, culturally responsive care partner support systems across Latin America.

### 4.3 Conclusion

This study provides a valuable and in-depth understanding of how family care partners in Lima, Peru, experience and respond to the BPSD in their daily caregiving. The findings reveal that managing BPSD represents a significant emotional and behavioural challenge for care partners and often tightens their relationship with the person living with dementia. However, care partners also develop flexible and adaptive strategies, such as changing communication styles, adapting the environment, and prioritising their emotional well-being, to navigate these challenges. This research contributes to the limited body of literature on dementia caregiving in Latin America, highlighting the importance of understanding caregiving experiences in diverse cultural contexts.

## Data Availability

The raw data supporting the conclusions of this article will be made available by the authors, without undue reservation.

## References

[ref1] Aguirre-Martens G. Salud en el Perú: De la cobertura en papel a la cobertura real. Peru: World Bank Blogs. (2023). Available online at: https://blogs.worldbank.org/en/latinamerica/healthcare-coverage-peru (Accessed March 7, 2025)

[ref2] Alcalde-Rabanal J. E. Lazo-González O. Nigenda G. (2011). Sistema de salud de Perú. Salud Publica Mex. 53, s243–s254. 21877089

[ref3] Almberg B. Grafström M. Winblad B. (1997). Major strain and coping strategies as reported by family members who care for aged demented relatives. J. Adv. Nurs. 26, 683–691. doi: 10.1046/j.1365-2648.1997.00392.x, 9354978

[ref4] Alzheimer’s Association (2023). 2023 Alzheimer’s disease facts and figures. Alzheimers Dement. 19, 1598–1695. doi: 10.1002/alz.13016 36918389

[ref5] Alzheimer’s Disease International. World Alzheimer Report 2009—Executive Summary. London, UK: Alzheimer’s Disease International; (2009) p. 24. Available online at: https://www.alzint.org/u/WorldAlzheimerReport-ExecutiveSummary.pdf (Accessed March 7, 2025).

[ref6] Ang C. S. Yuen A. Z. D. (2023). A qualitative study of dementia caregivers’ lived experiences in Singapore. Home Health Care Serv. Q. 42, 124–141. doi: 10.1080/01621424.2022.2164540 36594495

[ref7] Armstrong M. J. Alliance S. Corsentino P. Lunde A. Taylor A. (2022). Informal caregiver experiences at the end-of-life of individuals living with dementia with Lewy bodies: an interview study. Dementia 21, 287–303. doi: 10.1177/14713012211038428, 34340591

[ref8] Ayalon L. Ulitsa N. Nebowsky A. E. Schweda M. von Kutzleben M. (2025). Caring for an older person with dementia: behavioral problems in the eyes of family caregivers and migrant home care workers. Dementia 24, 884–901. doi: 10.1177/14713012251323661 40010380 PMC12171054

[ref9] Baharudin A. D. Din N. C. Subramaniam P. Razali R. (2019). The associations between behavioral-psychological symptoms of dementia (BPSD) and coping strategy, burden of care and personality style among low-income caregivers of patients with dementia. BMC Public Health 19:447. doi: 10.1186/s12889-019-6868-0, 31196141 PMC6565534

[ref10] Ballard C. Lowery K. Powell I. O’Brien J. James I. (2000). Impact of behavioral and psychological symptoms of dementia on caregivers. Int. Psychogeriatr. 12, 93–105. doi: 10.1017/S1041610200006840

[ref11] Bessey L. J. Walaszek A. (2019). Management of behavioral and psychological symptoms of dementia. Curr. Psychiatry Rep. 21, 1–66. doi: 10.1007/s11920-019-1049-5 31264056

[ref12] Boughtwood D. L. Adams J. Shanley C. Santalucia Y. Kyriazopoulos H. (2011). Experiences and perceptions of culturally and linguistically diverse family carers of people with dementia. Am. J. Alzheimers Dis. Other Dement. 26, 290–297. doi: 10.1177/1533317511411908, 21697144 PMC10845301

[ref13] Braun V. Clarke V. (2006). Using thematic analysis in psychology. Qual. Res. Psychol. 3, 77–101. doi: 10.1191/1478088706qp063oa

[ref14] Burley C. V. Casey A. N. Chenoweth L. Brodaty H. (2023). Views of people living with dementia and their families/care partners: helpful and unhelpful responses to behavioral changes. Int. Psychogeriatr. 35, 77–93. doi: 10.1017/S1041610222000849, 36330686

[ref15] Centro Nacional de Datos—CND. (2021). Información general del Perú. Available online at: https://cdn.www.gob.pe/uploads/document/file/1893016/Información%20general.pdf.pdf (Accessed March 7, 2025).

[ref16] Cheng S. T. (2017). Dementia caregiver burden: a research update and critical analysis. Curr. Psychiatry Rep. 19:64. doi: 10.1007/s11920-017-0818-2, 28795386 PMC5550537

[ref17] Crivelli L. Calandri I. L. Suemoto C. K. Salinas R. M. Velilla L. M. Yassuda M. S. . (2023). Latin American initiative for lifestyle intervention to prevent cognitive decline (LatAm-FINGERS): study design and harmonization. Alzheimers Dement. 19, 4046–4060. doi: 10.1002/alz.13101, 37204054 PMC11021182

[ref18] Custodio N. García A. Montesinos R. Escobar J. Bendezú L. (2008). Prevalencia de demencia en una población urbana de Lima-Perú: estudio puerta a puerta. An. Fac. Med. 69, 233–238.

[ref19] Custodio N. Lira D. Herrera-Perez E. del Prado L. N. Parodi J. Guevara-Silva E. . (2014). Informal caregiver burden in middle-income countries results from memory Centers in Lima—Peru. Dement. Neuropsychol. 8, 376–383. doi: 10.1590/S1980-57642014DN84000012 29213929 PMC5619187

[ref20] Custodio N. Soto-Añari M. Montesinos R. Caipa M. P. Ore-Gomez M. F. Rivera-Fernandez C. . (2022). Native American ancestry is associated with lower risk of Alzheimer’s disease: results from the genetic of Alzheimer’s disease in Peruvian populations (GAPP) study. Alzheimers Dement. 18:e066002. doi: 10.1002/alz.066002

[ref21] de Vugt M. E. Stevens F. Aalten P. Lousberg R. Jaspers N. Winkens I. . (2003). Behavioural disturbances in dementia patients and quality of the marital relationship. Int. J. Geriatr. Psychiatry 18, 149–154. doi: 10.1002/gps.807, 12571824

[ref22] Draper B. Finkel S. I. Tune L. Module 1—an introduction to BPSD. In: Draper B. Brodaty H. Finkel S. I., editors. The IPA Complete Guides to BPSD—Specialists Guide. Northfield: International Psychogeriatric Association; (2012). 1.1–1.16. Available online at: https://www.ipa-online.org/UserFiles/file/IPA_BPSD_Specialists_Complete_Guide_Online_2015_Final.pdf (Accessed March 7, 2025).

[ref23] Engel L. Loxton A. Bucholc J. Muldowney A. Mihalopoulos C. McCaffrey N. (2022). Providing informal care to a person living with dementia: the experiences of informal carers in Australia. Arch. Gerontol. Geriatr. 102:104742. doi: 10.1016/j.archger.2022.104742 35671552

[ref24] Feast A. Orrell M. Charlesworth G. Melunsky N. Poland F. Moniz-Cook E. (2016). Behavioural and psychological symptoms in dementia and the challenges for family carers: systematic review. Br. J. Psychiatry 208, 429–434. doi: 10.1192/bjp.bp.114.153684, 26989095 PMC4853642

[ref25] García J. R. Los tiempos del cuidado. El impacto de la dependencia de los mayores en la vida cotidiana de sus cuidadores. (2010). Instituto de Mayores y Servicios Sociales (IMSERSO). Available online at: https://sid-inico.usal.es/idocs/F8/FDO23622/Rogero_Garcia_10.pdf (Accessed October 20, 2024).

[ref26] Garcia-Ptacek S. Dahlrup B. Edlund A. K. Wijk H. Eriksdotter M. (2019). The caregiving phenomenon and caregiver participation in dementia. Scand. J. Caring Sci. 33, 255–265. doi: 10.1111/scs.12627, 30488971 PMC7432177

[ref27] GBD 2019 Dementia Forecasting Collaborators (2022). Estimation of the global prevalence of dementia in 2019 and forecasted prevalence in 2050: an analysis for the Global Burden of Disease Study 2019. Lancet Public Health 7, e105–e125. doi: 10.1016/S2468-2667(21)00249-8 34998485 PMC8810394

[ref28] Guba E. G. Criteria for assessing the trustworthiness of naturalistic inquiries. ECTJ. (1981);29:75–91

[ref29] Ibáñez A. Pina-Escudero S. D. Possin K. L. Quiroz Y. T. Peres F. A. Slachevsky A. . (2021). Dementia caregiving across Latin America and the Caribbean and brain health diplomacy. Lancet Healthy Longev. 2, e222–e231. doi: 10.1016/s2666-7568(21)00031-3 34790905 PMC8594860

[ref30] Ibañez A. Slachevsky A. Serrano C. Manual de buenas prácticas para el diagnóstico de demencias. Fundación INECO; (2020). Available online at: https://iberoamericamayores.org/2023/09/21/bid-ineco-manual-de-buenas-practicas-para-el-diagnostico-de-demencias/ (Accessed October 21, 2024).

[ref31] Ibanez A. Yokoyama J. S. Possin K. L. Matallana D. Lopera F. Nitrini R. . (2021). The multi-partner consortium to expand dementia research in Latin America (ReDLat): driving multicentric research and implementation science. Front. Neurol. 12:631722. doi: 10.3389/fneur.2021.631722 33776890 PMC7992978

[ref32] Instituto Nacional de Estadística e Informática—INEI. (2024). Población de la provincia de Lima supera los 10 millones 292 mil habitantes. Available online at: https://www.gob.pe/en/institucion/inei/noticias/894611-poblacion-de-la-provincia-de-lima-supera-los-10-millones-292-mil-habitantes (Accessed March 7, 2025)

[ref33] Isik A. T. Soysal P. Solmi M. Veronese N. (2019). Bidirectional relationship between caregiver burden and neuropsychiatric symptoms in patients with Alzheimer’s disease: a narrative review. Int. J. Geriatr. Psychiatry 34, 1326–1334. doi: 10.1002/gps.4965, 30198597

[ref34] Jorge C. Cetó M. Arias A. Blasco E. Gil M. P. López R. . (2021). Level of understanding of Alzheimer disease among caregivers and the general population. Neurolgia (Engl. Ed.) 36, 426–432. doi: 10.1016/j.nrleng.2018.03.004 34238525

[ref35] Kales H. C. Gitlin L. N. Lyketsos C. G. (2015). Assessment and management of behavioral and psychological symptoms of dementia. BMJ 350, 2–h369. doi: 10.1136/bmj.h369 PMC4707529 25731881

[ref36] Kaufer D. I. Cummings J. L. Ketchel P. Smith V. MacMillan A. Shelley T. . (2000). Validation of the NPI-Q, a brief clinical form of the neuropsychiatric inventory. J. Neuropsychiatry Clin. Neurosci. 12, 233–239. doi: 10.1176/jnp.12.2.233, 11001602

[ref37] Lazarus R. S. Folkman S. Stress, Appraisal, and Coping. 1st. New York, United States: Springer Publishing Company, Incorporated; (1984). Available online at: http://ebookcentral.proquest.com/lib/ki/detail.action?docID=423337 (Accessed April 29, 2025)

[ref38] Liu Z. Heffernan C. Tan J. (2020). Caregiver burden: a concept analysis. Int. J. Nurs. Sci. 7, 438–445. doi: 10.1016/j.ijnss.2020.07.012, 33195757 PMC7644552

[ref39] Lloyd J. Muers J. Patterson T. G. Marczak M. (2019). Self-compassion, coping strategies, and caregiver burden in caregivers of people with dementia. Clin. Gerontol. 42, 47–59. doi: 10.1080/07317115.2018.1461162, 29723129

[ref40] Malterud K. Siersma V. D. Guassora A. D. (2016). Sample size in qualitative interview studies: guided by information power. Qual. Health Res. 26, 1753–1760. doi: 10.1177/1049732315617444 26613970

[ref41] Meyer K. Rath L. Avent E. Benton D. Nash P. Wilber K. (2023). How do family caregivers of older adults cope with relationship strain? Aging Ment. Health 27, 1990–1999. doi: 10.1080/13607863.2023.2247353 37574858

[ref42] Montesinos R. Custodio B. Malaga M. Chambergo-Michilot D. Verastegui-Aranda G. Agüero K. . (2024). Influence of behavioral and psychological symptoms on caregiver burden for different types of dementia: clinical experience in Lima, Peru. Dement. Geriatr. Cogn. Disord. 53, 229–236. doi: 10.1159/000539335 38768581

[ref43] Nguyen H. Nguyen H. T. Nguyen N. B. Tran D. Harvey D. J. Nguyen B. T. . Testing the efficacy of a culturally adapted family dementia caregiver intervention (REACH VN): results from a cluster randomized controlled trial in northern Vietnam. Am. J. Geriatr. Psychiatry. (2024). doi: 10.1016/j.jagp.2024.10.011 PMC11903202 39547823

[ref44] Owokuhaisa J. Kamoga R. Musinguzi P. Muwanguzi M. Natukunda S. Mubangizi V. . (2023). Burden of care and coping strategies among informal caregivers of people with behavioral and psychological symptoms of dementia in rural South-Western Uganda. BMC Geriatr. 23:475. doi: 10.1186/s12877-023-04129-0, 37553634 PMC10408158

[ref45] Parra M. A. Baez S. Allegri R. Nitrini R. Lopera F. Slachevsky A. . (2018). Dementia in Latin America. Neurology 90, 222–231. doi: 10.1212/WNL.0000000000004897, 29305437 PMC5791795

[ref46] Prince M. Brodaty H. Uwakwe R. Acosta D. Ferri C. P. Guerra M. . (2012). Strain and its correlates among carers of people with dementia in low-income and middle-income countries. A 10/66 dementia research group population-based survey. Int. J. Geriatr. Psychiatry 27, 670–682. doi: 10.1002/gps.2727, 22460403 PMC3504977

[ref47] Rathnayake S. Jones C. Calleja P. Moyle W. (2019). Family carers’ perspectives of managing activities of daily living and use of mHealth applications in dementia care: a qualitative study. J. Clin. Nurs. 28, 4460–4470. doi: 10.1111/jocn.15030, 31408554

[ref48] Ruiz-Calderón R. Palomares-Cristóbal R. Situación de la Población Adulta Mayor, Trimestre: Julio-Agosto-Setiembre 2024. Perú: Instituto Nacional de Estadística e Informática; (2024) p. 50. Available online at: https://m.inei.gob.pe/media/MenuRecursivo/boletines/boletin_adulto_mayor_3t24.pdf (Accessed March 7, 2025).

[ref49] Sachdev P. S. Blacker D. Blazer D. G. Ganguli M. Jeste D. V. Paulsen J. S. . (2014). Classifying neurocognitive disorders: the DSM-5 approach. Nat. Rev. Neurol. 10, 634–642. doi: 10.1038/nrneurol.2014.181, 25266297

[ref50] Shenton A. K. (2004). Strategies for ensuring trustworthiness in qualitative research projects. Educ. Inf. 22, 63–75. doi: 10.3233/EFI-2004-22201

[ref51] Sosa A. L. Brucki S. M. Crivelli L. Lopera F. J. Acosta D. M. Acosta‐Uribe J. . (2024). Advancements in dementia research, diagnostics, and care in Latin America: highlights from the 2023 Alzheimer's Association international conference satellite symposium in Mexico City. Alzheimers Dement. 20, 5009–5026. doi: 10.1002/alz.13850, 38801124 PMC11247679

[ref52] The World Bank. (2025). World Bank country and lending groups. Available online at: https://datahelpdesk.worldbank.org/knowledgebase/articles/906519-world-bank-country-and-lending-groups (accessed March 7, 2025)

[ref53] Tong A. Sainsbury P. Craig J. (2007). Consolidated criteria for reporting qualitative research (COREQ): a 32-item checklist for interviews and focus groups. Int. J. Qual. Health Care 19, 349–357. doi: 10.1093/intqhc/mzm042, 17872937

[ref54] Tu J. Li H. Ye B. Liao J. (2022). The trajectory of family caregiving for older adults with dementia: difficulties and challenges. Age Ageing 51:afac254. doi: 10.1093/ageing/afac254 36469090

[ref55] Wimo A. Gauthier S. Prince M. Global Estimates of Informal Care. London: Alzheimer’s Disease International (ADI); (2018). Available online at: https://www.alzint.org/u/global-estimates-of-informal-care.pdf (Accessed April 14, 2025).

[ref56] World Health Organization. Global Status Report on the Public Health Response to Dementia. Geneva: World Health Organization; (2021). p. 137. Available online at: https://iris.who.int/server/api/core/bitstreams/9e8aab50-ca34-45e0-9fb0-26e675746f65/content (Accessed March 7, 2025)

