# Supplementary material for: Navigating dementia care: a qualitative study of family care partners’ experiences in managing behavioural and psychological symptoms in Lima, Peru
Source: Front Dement. 2026 Mar 16;5:1774816. doi: 10.3389/frdem.2026.1774816 (PMC13033498; doi:10.3389/frdem.2026.1774816)
Supplement: Supplementary file 1 [file Supplementary_file_1.docx]

Supplementary Material

# Interview Guide

## Initial questions

- Who do you care for?
- How old is your relative? (person living with dementia)
- How long has your relative had the diagnosis?
- How old are you? (care partner)
- Which gender do you identify with?
- How long have you been a care partner for your relative?

## General Experience and Challenges of Caring for a person living with dementia

- How would you describe your experience as a care partner for a person with dementia?
- What have been the most challenging aspects of managing your family member’s behavioural symptoms? And why?

## Strategies for Managing the Behavioural Symptoms

- How do you manage the behavioural symptoms of the disease? How did you realise this worked for your family member?
- Can you describe a situation where managing the behavioural symptoms worked well?
- Can you tell me about a situation where something did not work in managing behavioural symptoms?
- Can you describe whether the strategies have helped you, and in what way they have helped you?

## Strategies, Support and Resources

- What type of support (family, friends, healthcare professionals) do you have for managing these symptoms?
- Have you received any formal training or advice on how to handle these behaviours? How has it helped you?

# Example of the analysis process

## Example of the data analysis:

| **Data** | **Code** | **Theme** |
| --- | --- | --- |
| She would get very anxious, she would cry and shout at me […] and I felt very sad about it, and I also didn't know how to manage at that time, eh? And well, that made me feel very, very frustrated | Painfulness of BPSD | Caregiving as an emotional and behavioural challenge |
| I realised, that if the caregiver starts a discussion with a person with dementia, he/she has already lost. The battle is already lost. | Arguing is a lost battle | The role of adaptation and flexibility when managing the BPSD |
| "My best therapy: the gym," because it helped me de-stress. | Importance of selfcare | Building the care partner’s emotional well-being to support caring |

## A mind map created to build categories:

**
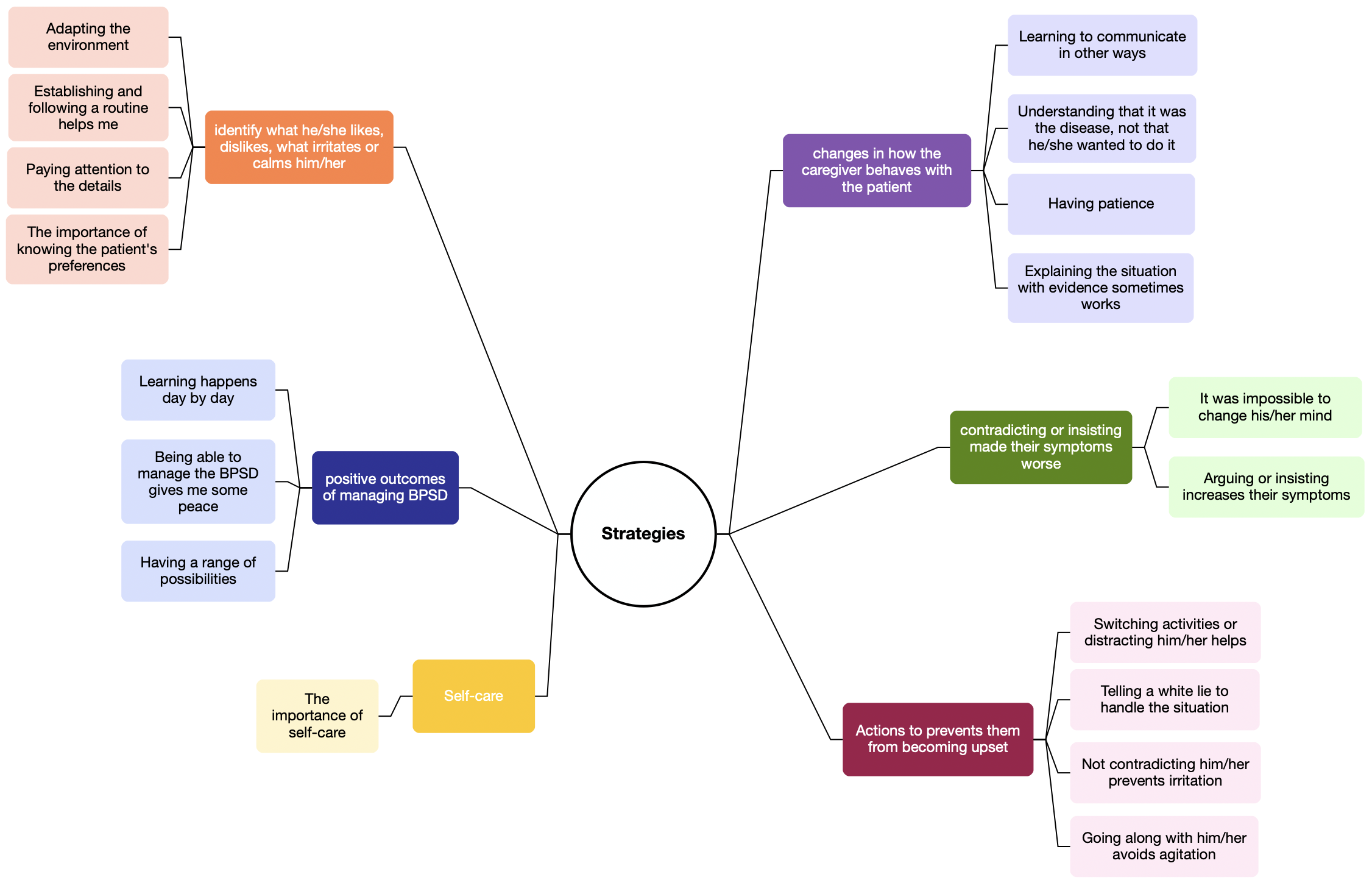
**
